# Retrospective analysis of children diagnosed with Kawasaki disease

**DOI:** 10.55730/1300-0144.5662

**Published:** 2023-02-02

**Authors:** Fatih VAROL, Reyhan DEDEOĞLU, Aziz KILIÇ, Murat T. BAKAR, Amra ADROVİC, Sezgin ŞAHİN, Mehmet YILDIZ, Kenan BARUT, Halit ÇAM, Özgür KASAPÇOPUR

**Affiliations:** 1 Department of Pediatric Intensive Care, University of Health Science Sancaktepe Şehit Profesör Dr. İlhan Varank Training and Research Hospital, İstanbul, Turkiye; 2 Department of Pediatric Cardiology, Cerrahpaşa Medical School, İstanbul University-Cerrahpaşa, İstanbul, Turkiye; 3 Department of Pediatrics, Faculty of Medicine, Ankara University, Ankara, Turkiye; 4 Department of Public Health, Medical School, Marmara University, İstanbul, Turkiye; 5 Department of Pediatric Rheumatology, Cerrahpaşa Medical School, İstanbul University-Cerrahpaşa, İstanbul, Turkiye

**Keywords:** Kawasaki, intravenous immunoglobulin, coronary aneurysm

## Abstract

**Background/aim:**

The aim of our study was to evaluate the long-term impacts of Kawasaki disease on our patients regarding coronary involvement demographic characteristics, treatment regimens, and clinical course.

**Materials and methods:**

Our study included 104 patients diagnosed and hospitalized with Kawasaki disease in our center, from January 2004 to January 2019. In our study, patients were divided into three groups according to coronary artery involvement. Patients in group 1 had no echocardiographic findings, while the ones in group 2 had coronary artery dilatation and ones in group 3 had coronary artery aneurysm (CAA).

**Results:**

Among 104 patients, the median age was 9.15 (3.0–22.0) years, and 61 of the patients were male while 43 of the patients were female. With a wide range of 1.50–16.50 years of follow-up time, the median diagnosis age of our patients was 31 months (3.0–164.0). Fever duration (median day 10 (5–21), p = 0.025) was statistically significantly higher in group 3. Blood C-reactive protein (CRP) levels, white blood cell (WBC) counts, and neutrophil counts were significantly higher in group 3. There was a statistically significant difference between patients in group 3 and group 2 in which the lowest strain deformation values were in the patients of group 3. In contrast to group 1, the time for initiation of IVIG therapy is significantly prolonged both in group 2 (median: 9.5 days, p = 0.028) and group 3 (median: 10 days, p = 0.036).

**Conclusion:**

In our study, serum CRP levels, WBC count, and neutrophil count were higher in patients with coronary artery abnormalities, in agreement with the previous studies. In the light of our results, we consider that the most important determining factor for the development of coronary artery aneurysm is the time of intravenous immunoglobulin (IVIG) administration.

## 1. Introduction

Kawasaki disease is an acute vasculitis of medium-sized vessels during childhood, and it especially affects coronary arteries. The disease was first reported by Kawasaki of Japan in 1967 [1,2]. Although it is a self-limiting disease, almost 25% of patients suffer from coronary artery aneurysms if not treated properly with early high-dose IVIG therapy [3]. The most common cardiac complications caused by coronary artery damage are myocardial ischemia and infarction [4]. It may also lead to myocardial fibrosis, valvulitis and aortic root dilatation, and sudden death [5,6]. The main focus of early-stage treatment of Kawasaki disease is to suppress inflammation in coronary arteries and to prevent complications such as coronary thrombosis and aneurysms [7]. The primary treatment for Kawasaki disease is early high-dose IVIG therapy with aspirin conventionally used as an antiinflammatory and antithrombotic agent [7,8]. According to the Scientific Statement of the American Heart Association (AHA) in 2017, IVIG treatment significantly decreases coronary complications, from 25% to 4% [3].

Speckle tracking echocardiography (STE) is a recent two-dimensional echocardiography method for detecting ventricular dysfunction. Myocardial deformation (strain) is defined as the relative change of muscle from its original length. Strain imaging provides the analysis of the region of interest, with information about the regional myocardial function and also global function (global and regional strain) [9].

Several studies are exhibiting the relationship between demographic characteristics, clinical course, and coronary artery involvement-affecting cardiac functions. However, data regarding the long-term outcomes of the disease is limited. In our study, we aimed to investigate the long-term impacts of the disease on our patients, regarding coronary involvement demographic characteristics, treatment regimens, and clinical course.

## 2. Methods

Patients have given their informed consent for participation in the research study during data collection. Approvals were received by the İstanbul University, Faculty of Medicine – Cerrahpaşa, Clinical Ethics Committee before the beginning (protocol number: 83045809-604.01.02-, 02.05.2017). The study has been conducted in accordance with the principles outlined in the Helsinki Declaration.

### 2.1. Study population and diagnostic criteria

In our study we assessed 104 patients diagnosed and hospitalized with Kawasaki Disease in the pediatric emergency, rheumatology, and cardiology departments of İstanbul University, Faculty of Medicine – Cerrahpasa, from January 2004 to January 2019.

Among all who are diagnosed or suspected with Kawasaki disease from 2004 to 2019 in the pediatric emergency department, cases not meeting the diagnostic criteria of AHA 2017 were excluded from the study [3]. Classical Kawasaki disease was diagnosed with at least 4 of the 5 principal clinical features (erythema and cracking of lips, strawberry tongue, and/or erythema of oral and pharyngeal mucosa, bilateral bulbar conjunctival injection without exudate, maculopapular, diffuse erythroderma, or erythema multiforme-like rash, erythema and edema of the hands and feet in the acute phase and/or periungual desquamation in the subacute phase, cervical lymphadenopathy (≥1.5 cm diameter, usually unilateral) in patients who had a fever lasting at least 5 days^3^. Patients with fever lasting at least 5 days and 2 or 3 compatible clinical criteria and infants with fever lasting at least 7 days without other explanation were diagnosed with incomplete Kawasaki disease if supportive laboratory tests or echocardiographic findings were present [3].

### 2.2. Demographics and laboratory findings

Our data were retrospectively recorded through a questionnaire which included patient name, sex, and age, date of disease onset, date of diagnosis, date of presentation, clinical symptoms, echocardiographic findings, laboratory tests, therapy, and prognosis. Sources of information were either parents or patients themselves. Also, we collected clinical information and laboratory results from recorded files.

### 2.3. Echocardiography

#### 2.3.1. Standard transthoracic echocardiography

We used a commercially available echocardiography machine (Philips iE33, Philips Medical Systems) for transesophageal echocardiography (TTE) in the Pediatric Cardiology Department of Cerrahpaşa Medical Faculty. Doppler scan recordings were used for detecting left ventricular (LV) diastolic functions.

E-wave expressed peak early diastolic velocity, peak late diastolic velocity, and A-wave expressed atrial filling. The E-wave is larger than the A-wave in normal diastolic function, the E/A ratio will fall because of the increasing atrial filling wave (A-wave) in impaired relaxation [10].

#### 2.3.2. Speckle tracking echocardiography (STE)

**Figure 1A:**
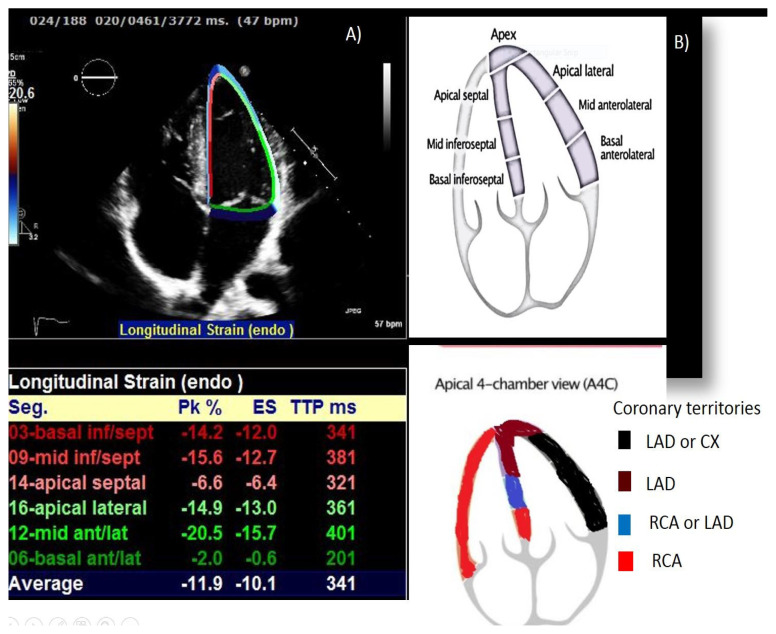
Stain measurements of speckle tacking echocardiography in 4-chamber view. Figure 1B: Supplying segments of coronary arteries on 4-chamber view.

STE is a new technique that analyzes motion, by tracking natural acoustic reflections within an ultrasonic window and assessing ventricular deformation (strain) at the regions of interest by the observer. The presence of myocardial disease could be detected most sensitively with longitudinal LV mechanics [11]. We decided to present global longitudinal strain (GLS) with STE from cardiac cycles, which is expressed as global strain curves generated from each view and recorded as a percentage: GLS 4ChS (%), GLS 3ChS (%), GLS 2ChS (%) (Figures 1A and 1B).

### 2.4. Patient groups

We assessed luminal dimensions normalized for BSA as Z-scores according to Dallaire et al. [12]. We determined a mean Z-score < 2.0 SD as normal, whereas a mean Z-score between 2.0 and < 2.5 SD was presented as dilation. Small aneurysm of coronary arteries was defined as a mean Z-score 2.5 to < 5.0, medium aneurysm 5.0 to < 10, and large aneurysm from ≥ 10 or absolute dimension ≥ 8 mm. Given the inflammatory process and possible aneurysms of coronary arteries affecting LV segments, we divided Kawasaki patients into subgroups by coronary involvement during the follow-up as coronary dilatation and coronary aneurysms, and unaffected coronary arteries. We compared these subgroups concerning affected cardiac segments, follow-up time, disease severity, medications used, demographics, and laboratory indices. Patients in our study were invited to our clinic, and control echocardiography was performed for assessing long-term effects and outcomes. Patients were divided into three groups according to coronary artery involvement. Patients in group 1 had no echocardiographic findings, while the ones in group 2 had coronary artery dilatation and ones in group 3 had coronary artery aneurysm.

### 2.5. Statistical analysis

Statistical analyses were done by using Statistical Package for the Social Sciences (SPSS) statistical software for Windows, 20.0. Numbers, frequencies (%), ratios, medians, and range (min-max) were used in the descriptive statistics of the data. The distribution of variables was checked with the Kolmogorov Smirnov test. In the analysis of quantitative data, t-test and Mann-Whitney U tests were used. The χ^2^ test was used to compare categorical variables, Fisher test was used when chi-square conditions could not be satisfied. The effect levels were investigated by logistic regression analyses. Variables with a p ≤ 0.05 using logistic regression analyses were accepted as independent risk factors. The OR and 95% CI for each risk factor were determined. Results were evaluated with a 95% confidence interval and with a significance level of p < 0.05.

## 3. Results

### 3.1. Demographics and study groups

Among 104 patients, the median age was 9.15 (3.0–22.0) years, and 61 of the patients were male while 43 of the patients were female. With a wide range of 1.50–16.50 years of follow-up time, the median diagnosis age of our patients was 31 months (3.0–164.0). There was no statistically important difference between the three groups considering age and sex (Table 1). The risk for developing coronary arterial dilatation and aneurysm was found to be statistically significantly higher (p = 0.014) in patients with chronic diseases. Coronary Z scores of subgroups were presented on tables (Table 2).

### 3.2. Clinical findings

Fever durations (median day 10 (5–21), p = 0.025) were statistically significantly higher in group 3 (Table 1). Among all other symptoms, conjunctivitis was the only statistically significant symptom (p = 0.029) for the development of coronary artery dilatation and aneurysm (Table 2).

### 3.3. Laboratory findings

When we compared the data of our patients, there was a statistically significant difference in blood CRP levels on hospitalization day (median value of group 1 is 3.3 (0.1–33.7), of group 2 is 8.83 (0.3–27), p = 0.041) and fever duration days (median value of group 1 is 7 (5–30), of group 2 is 10 (5–17), p = 0.011) between group 1 and group 2 (Table 1).

Similarly, according to the comparison of laboratory parameters in group 1 and group 3, WBC counts (median value of group 1 is 13.250 (6.500–19.9000), of group 3 17.300 (8.700–32.400), p = 0.042) and neutrophil counts (median value of group 1 is 8.050 (1.400–32.600), of group 3 is 12.200 (4.100–26.700), p = 0.014) were statistically significantly higher in group 3 (Table 1).

When we consider the median sodium values in one-way ANOVA analysis, group 3 had higher median sodium levels than group 1 and group 2.

### 3.4. Echocardiographic findings

Comparison of LV strain and cardiac functions of Kawasaki patients according to the coronary artery involvement by STE.

Global LV systolic and diastolic functions, which had been assessed with conventional echocardiographic methods, were in normal ranges in all patients. There was no difference between subgroups (Table 3).

All mean STE and global strain values of 17 segments in the left ventricle are summarized in Table 4.

In our study, we found lower strain values at mid inferior septum, basal inferior septum, and apical septum in group 3 patients compared to patients in groups 1 and 2. Also, there was a statistically significant difference between patients in group 3 and group 2 in which the lowest strain deformation values were in the patients of group 3 (Table 3, Figure).

### 3.5. Management modalities

As seen in Table 2, in a total of 104 patients, once 2 g/kg, twice 1 g/kg, and twice 2 g/kg IVIG therapy regimens were applied to 93, 4, and 7 of them, respectively. The median onset day of IVIG therapy is 8 (5.0–30.0). In contrast to group 1, the time for initiations of IVIG therapy is significantly prolonged both in group 2 (median: 9.5 days, p = 0.028) and group 3 (median: 10 days, p = 0.036) (Table 1). However, there was not a significant difference in the IVIG time between group 2 and group 3.

## 4. Discussion

The median diagnosis age of our patients was 31 months. Previous studies assessed that most of the patients (80%) diagnosed with Kawasaki disease are younger than 5 years, with a peak in late infancy [13]. There are few studies stating that the risk of coronary artery aneurysms increases with low age in children with Kawasaki disease [14]. We did not find a statistically significant difference between age and coronary artery aneurysms in our patients. Also, lots of studies evaluated the relationship between sexes and disease complications. Some of them resulted in a higher risk of complications in male patients [15,16]. Contrary to these, we found no statistically significant sex-specific risk for dilation and aneurysm. In our opinion, this result is due to the similarity of our patients’ symptom duration and treatment initiation time.

In our study, conjunctivitis is the only statistically significant symptom related to coronary artery abnormalities. In several previous studies, conjunctivitis was declared as one of the most common symptoms in the clinical course of Kawasaki disease; there is no study in the literature indicating the relationship between conjunctivitis and the development of coronary artery abnormalities.

Our data showed that higher serum CRP levels greatly increase the risk for coronary artery dilatation, as shown in Table 1; however, we found no statistically significant evidence for serum CRP levels considering the development of coronary artery aneurysms. In the literature, serum CRP levels were found as a risk factor for the development of coronary artery dilatations and aneurysms in Kawasaki disease. As an acute-phase reactant protein, CRP indicates injury, infection, and inflammation [17]. The degree of inflammation during the acute phase of the illness is correlated with CRP levels [18]. Kobayashi et al. showed that a high CRP level was an independent risk factor for IVIG-unresponsiveness and coronary artery aneurysms [19]. Xie et al. evaluated a 1.913 odds ratio for CRP > 14 mg/dL for the development of coronary artery abnormalities in Kawasaki disease [20]. Similarly, Mori et al. described higher CRP levels are associated with IVIG resistance and coronary artery aneurysm [21]. In a recent study, CRP≥13 mg/dL was found as a predictor of coronary artery abnormality [22]. A Chinese study exhibited that 1 mg/dL increase in CRP levels is associated with a 0.02-fold increase in risk for coronary artery abnormalities [23]. Contrarily, a study investigating the biomarkers for Kawasaki disease and the development of coronary artery aneurysms showed that there is no correlation between CRP levels and CAA [24]. Also, another study indicates that there is no statistical association between the level of erythrocyte sedimentation rate (ESR), CRP, and platelet (PLT) with late cardiac involvement [25]. Additionally, a study from China reported that decreased Annexin A1 levels are directly associated with coronary artery aneurysms. Even though there is a relationship between Annexin A1 and CRP, blood CRP levels are not directly correlated with coronary artery aneurysm development [26].

In our study, one of the factors increasing the risk of coronary artery aneurysm was the white blood cell (WBC) count. Our results showed that the median WBC count in patients with coronary artery aneurysm was higher than the patients with no coronary artery abnormalities. Similarly, a study from China exhibited that the maximal leukocyte counts of the patients with cardiac sequelae were higher than in those without sequelae. In addition, they confirmed that the proportion of patients with cardiac sequelae increased with maximal leukocyte counts in every age group [27]. According to another study, the WBC count was higher in the subacute phase of Kawasaki disease [28]. As previously reported by Mori et al., WBC count was higher in 87, 5% of children with coronary artery abnormalities after IVIG therapy. Moreover, in multiple logistic regression analysis, CRP levels, neutrophil count, and WBC count can be used as predictors for the development of coronary artery abnormalities in Kawasaki disease [21]. A previous study assessed that patients with Kawasaki disease shock syndrome either have a higher risk of developing coronary artery aneurysms and higher blood CRP levels, WBC, and neutrophil counts [29]. In a metaanalysis, among several risk factors, neutrophil count demonstrated a large effect size as a predictive risk factor [30]. In agreement with these studies, in our study, these 3 parameters were found statistically significant in patients with coronary artery abnormalities.

In this study, strain values at the mid inferior septum, basal inferior septum, and apical septum in patients with coronary aneurysm were lower compared to patients without coronary aneurysm, dilatation and the statistically significantly lowest strain deformation values were found in coronary aneurysm group. Similarly, in a previous study of our team [6], we detected subclinical myocardial compromise of past Kawasaki disease by STE. Our results indicated that the decline in strain measurements reflects myocardial dysfunction early before systolic dysfunction occurs. Also, we tried to find out if the subclinical myocardial dysfunction is limited or preceded by a particular region of coronary arteries. We compared past Kawasaki patients and controls (healthy children) and found that basal inferioseptal, basal anterolateral, apical septal, and apical inferior segments had lower STE than controls. Nevertheless, we could not find a significant difference between the left ventricular segments of patients with or without coronary lesions. We commented on this result because of the small number of past Kawasaki patients with coronary aneurysm for statistical analyses.

In conclusion, with this study, we found a significantly lower risk of development of coronary artery dilatation and aneurysms in patients with early IVIG treatment while our median onset day of IVIG therapy was 8. In fact, we believe that patients recognized to have Kawasaki disease should also be given treatment without waiting for 7–8 days. Nevertheless, our center is a tertiary clinic for Kawasaki patients and mostly, patients are sent to our center for long-lasting fever; therefore, we had many patients that were not recognized and had delayed IVIG treatment. In the management of Kawasaki patients, the major determinant of the best outcome appears to be the timing of IVIG therapy. The AHA recommends treating Kawasaki patients with IVIG ideally within 7 days but treating within 10 days of onset of illness is also recommended^7^. As American Heart Association declares, intravenous immunoglobulin treatment is crucial, and it is the most important factor affecting coronary complications. As we agree on this, there is a significant need for emphasizing the onset day of IVIG treatment during the clinical course of Kawasaki disease. Historically Newburger’s 1986 trial managed treatment within 10 days while the first RCT of Furusho’s required within 7 days of the beginning of the disease [8,31]. In a study from Japan in which the national database was used, the authors compared 75 patients treated with IVIG between 11 and 20 days with counterparts of patients treated between 4 and 8 days. Half of the patients in the 11 to 20 days group developed coronary artery abnormalities during the treatment but half of these get normalized coronary arteries 1 month after the disease [32]. Bal et al. showed that patients receiving IVIG therapy within 10 days of onset of clinical symptoms have a lower risk of developing cardiac complications [33]. While evaluating the risk factors for developing coronary artery abnormalities, Sabharwal et al. found that only the variables associated with a longer duration of fever can predict coronary artery abnormalities [34]. In accordance with this study, another study from Korea reported that the risk of coronary artery involvement increases proportionally with the duration of total fever [35]. On the other hand, in a previous study, the duration of fever in patients who received treatment before the 10th day did not have a significant difference compared to the patients who received it after the 10th day [36]. Similar to the general opinion in the literature, our patients with prolonged fever are more likely to develop coronary artery abnormalities, and this supports our conclusion for early IVIG therapy.

Although there are studies suggesting a combination of IVIG and steroid therapy might prevent coronary artery abnormalities [37], in our study, 30 patients received steroid therapy for persistent fever and there is no statistically significant difference in the clinical course of patients for the development of coronary artery complications.

We have some limitations in this study. We had not performed deformation imaging in all patients (strain) at the beginning of the disease because of the lack of presence of this method at the time. Because our clinic is a tertiary center and patients are referred to us long after the beginning of fever and sometimes suspicions of Kawasaki disease, we could not administer medications to the patients in a standard manner and had to compare the management regimens lately.

In conclusion, in agreement with the previous studies, serum CRP level, WBC count and neutrophil count were higher in patients with coronary artery abnormalities. In the light of our results, we consider that the most important determining factor for the development of coronary artery aneurysm is the time of IVIG administration. Although it is not directly related to the involved coronary artery, we recommend the use of echocardiography in long-term follow-up of the affected cardiac segments.

## Figures and Tables

**Table 1:** Demographic and laboratory findings of the patients with Kawasaki disease.

	Unaffected coronary arteries Median (IQR), n = 70	Coronary dilatation (Z-score 2.1 ± 1.12) Median (IQR), n = 18	Coronary aneurysm (Z-score 3.6 ± 1.56) Median (IQR), n = 16
Age†	9 (7.425)	8.75 (4.650)	10 (3.725)
Diagnosis age†	30 (41.500)	25.5 (45.000)	37.5 (46.500)
Follow-up time (day)†	6 (5.625)	5.5 (5.500)	5.5 (5.875)
IVIG day *§	7 (5.000)	9.5 (4.500)	10 (4.750)
AST (U/L)†	30 (21.000)	31.5 (39.000)	25 (18.000)
ALT (U/L)†	23 (27.000)	28 (44.250)	19 (17.250)
ESR 3 (mm/h)†	32 (21.500)	32 (26.250)	32 (35.500)
ESR 4 (mm/h)†	14 (10.000)	14.5 (13.750)	17 (11.500)
CRP 1 (mg/dL)*	3.3 (7.2125)	8.83 (7.775)	4.1 (4.610)
CRP 2 (mg/dL)†	1.1 (2.0975)	1.95 (2.125)	0.4 (2.430)
CRP 3 (mg/dL)†	0.2 (0.345)	0.55 (0.7275)	0.17 (0.6025)
LDH (U/L) †	258,5 (139.250)	235 (88.500)	306 (114.000)
WBC count§ (/mm^3^)	13250 (8750)	15100 (7675)	17300 (15150)
Neutrophil count (/mm^3^)§	8050 (7225)	8950 (10650)	12200 (7700)
Lymphocyte count (/mm^3^)†	3.950 (2.725)	4.300 (5.075)	3.900 (3600)
Neutrophil to Lymphocyte Ratio†	1.7 (1.865)	2.4 (4.840)	2.5 (3.640)
Platelet count 1 (/mm^3^)†	332.000 (245250)	375.000 (187000)	499.000 (427750)
Platelet count 2 (/mm^3^)†	479.000 (240000)	524.000 (268250)	523.000 (190750)
Platelet count 3 (/mm^3^)†	320.000 (254000)	346.000 (157750)	370.500 (99000)
Fever day*§	7 (5.000)	10 (5.500)	10 (4.750)

IVIG: intravenous immunoglobulin, AST: aspartate aminotransferase, ALT: alanine aminotransferase, ESR: erythrocyte sedimentation rate, CRP: C-reactive protein, LDH: lactate dehydrogenase, WBC: white blood cell.

C-reactive protein 1 refers to C-reactive protein value on admission, C-reactive protein 2 refers to C-reactive protein value 24 h after the IVIG treatment, and C-reactive protein 3 refers to C-reactive protein value 48 h after the IVIG treatment.

*: p ≤ 0.05 level for patients with unaffected coronary arteries and patients with coronary artery dilatation.

†: The statistical difference was not significant (p > 0.05) between the 3 groups of Kawasaki disease.

§: The statistical difference was significant for patients with unaffected coronary arteries and coronary aneurysm (p ≤ 0.05).

**Table 2:** Comparison of clinical findings and treatment regimens of the patients with Kawasaki disease.

	Unaffected coronary arteries	Coronary dilatation/aneurysm	p

Sex†			
Male	40	21	0.653
Female	30	13	

Chronic disease§			
Yes	14	16	0.004
No	56	18	

Chronic medicine†			
Yes	8	6	0.378
No	62	28	

IVIG dosage†			
Once 2 g/kg	66	27	0.039
Twice 1 g/kg	2	2	
Twice 2 g/kg	2	5	

Aspirin day†			
<3 months	26	5	0.019
>3 months	44	29	

Steroid†			
Yes	19	11	0.582
No	51	23	

Conjunctivitis§			
Yes	44	27	0.089
No	26	7	

Lymphadenopathy†			
Yes	40	19	0.903
No	30	15	

Rash†			
Yes	51	26	0.693
No	19	8	

BCG scar†			
Yes	11	3	0.541
No	59	31	

Strawberry tongue†			
Yes	46	25	0.422
No	24	9	

Cracked lips†			
Yes	58	27	0.670
No	12	7	

Arthritis†			
Yes	17	8	0.933
No	53	26	

Peeling†			
Yes	49	24	0.951
No	21	10	

Abdominal pain†			
Yes	14	7	0.944
No	56	27	

Vomiting†			
Yes	12	6	0.949
No	58	28	

Diarrhea†			
Yes	8	6	0.378
No	62	28	

Type†			
Complete	48	24	0.834
Incomplete	22	10	

†: The statistical difference was not significant (p > 0.05) for coronary artery abnormalities (−) versus unaffected coronary in the Kawasaki disease group.

§: The statistical difference was significant (p < 0.05) for coronary artery abnormalities (+) versus unaffected coronary in the Kawasaki disease group.

§: p > 0.05 level for Kawasaki patients with unaffected coronary arteries, and patients with coronary abnormalities.

**Table 3:** Comparison of the echocardiographic and pulse wave tissue Doppler findings between Kawasaki patients with CAA, CAD, and without coronary artery involvement.

	Unaffected coronary arteries, mean ± SD (min–max)	Coronary dilatation, mean ± SD (min–max)	Coronary aneurysm, mean ± SD (min–max) (+)	p
Ejection fraction†	65.6 ± 4.3 (60–76)	65.5 ± 3.7 (60–71)	70.3 ± 3.3 (63–74)	**^0.058^**
Shortening fraction†	35.6 ± 3.6 (31–44.3)	35.4 ± 3.1 (31–40.5)	41.1 ± 3.1 (37–46)	**^0.09^**
Trans mitral E-wave†	89.7 ± 20.9 (10–119)	91.2 ± 11.2 (73–114)	95.7 ± 13.5 (77–118)	**^0.079^**
Trans mitral A-wave†	54.5 ± 14.7 (5.6–86)	61.7 ± 14.9 (45–92)	57.5 ± 7.8 (45–69)	**^0.067^**
The ratio of E/A waves†	1.6 ± 0.2 (1.3–2.2)	1.5 ± 0.3 (0.9–2)	1.6 ± 0.2 (1.2–2.1)	**^0.486^**
**Pulsed-wave tissue Doppler**				
Mitral lateral E’ wave†	19.4 ± 3.3 (14–27.1)	19.8 ± 3.8 (15.7–27)	20 ± 4.2 (13.2–26.5)	**^0.971^**
Mitral lateral A’ wave†	8.1 ± 2.3 (3.9–13.4)	8.2 ± 2.5 (6.4–14.3)	9 ± 3.4 (4.1–14.7)	**^0.813^**
Mitral lateral S’ wave†	10.9 ± 2 (7.3–15.1)	9.2 ± 3.5 (1–12.5)	11.9 ± 2.2 (9.1–15)	**^0.732^**
E/E’†	4.9 ± 0.8 (3.7–6.9)	4.6 ± 1.1 (3.5–7.2)	5 ± 1.4 (3.3–7.5)	**^0.072^**
Right ventricle FAC†	44 ± 9.3 (22–60)	45.2 ± 8.2 (32–57)	46.2 ± 8.1 (34–58)	**^0.06^**
Tapse^*^	21.3 ± 2.7 (16–27)	21.7 ± 4.4 (15–29)	20.8 ± 4.2 (13.2–29)	**^0.372^**
Tricuspid E’ wave†	14.4 ± 3.8 (10–24.5)	14.7 ± 1.9 (11.3–18.5)	12.5 ± 4 (5.6–17.9)	**^0.126^**
Tricuspid A’ wave†	9.2 ± 2.5 (6–13.8)	7.6 ± 2.4 (5.1–12.5)	8.6 ± 2.9 (5.2–14.1)	**^0.092^**
Tricuspid S’ wave†	12.8 ± 2.4 (8.4–17)	12.9 ± 1.9 (11–17.5)	13.4 ± 3.5 (7.1–17.4)	**^0.137^**

RV; right ventricle, RV FAC; right ventricle fractional area change, Tapse; tricuspid annular plane systolic excursion (normal value > 18 mm), the early, rapid filling phase of diastole is represented by the E-wave. Atrial contraction occurs in late diastole, and is represented by the A-wave. The early diastolic tissue Doppler velocity, commonly denoted as E’, represents right ventricular relaxation. Under normal conditions, the E velocity is greater than A velocity. As the ventricle becomes less compliant, the E velocity decreases, and the E/A ratio lowers.

†: The statistical difference was not significant (p > 0.05) for coronary aneurysm (−) versus coronary dilatation in the Kawasaki disease group.

§: p > 0.05 level for Kawasaki patients with unaffected coronary arteries and patients with coronary aneurysm.

**Table 4:** Strain measurements with speckle-tracking echocardiography data of the patients with Kawasaki disease.

17-segment model (AHA)	Unaffected coronary arteries, mean ± SD (min–max)	Coronary dilatation, mean ± SD (min–max)	Coronary aneurysm, mean ± SD (min–max) (+)	p
*Four-chamber view longitudinal strain (%)*				
Basal inferior septal*§	20.6 ± 4.2 (12–27.2)	19.7 ± 2.3 (16.7–24)	18.8 ± 5.2 (12.5–31)	0.551
Mid inferior septal*§	18.9 ± 2.7 (12.2–22.5)	19.8 ± 2.6 (16–24)	16.8 ± 2.5 (12.7–19)	0.068
Apical septal*§	19.8 ± 2.7 (14.7–24.2)	20.5 ± 4.3 (16–29)	17 ± 3.5 (12–22)	0.028
Basal anterolateral†	19 ± 5 (6–27.2)	20.4 ± 3.7 (15–27)	21.3 ± 8.5 (10–38.2)	0.592
Mid anterolateral†	16.4 ± 5 (11–35)	17.4 ± 2.5 (13.3–22)	14.5 ± 4 (9.3–21.7)	0.396
Apical lateral†	16.8 ± 6.9 (6.7–39)	18 ± 4.3 (12–24)	14.5 ± 4.7 (7.5–23.5)	0.491
GLS 4ChS (%)†	18.4 ± 3 (13–27.8)	19.5 ± 2.5 (16–23.2)	17.5 ± 3.7 (11.7–24.4)	0.420
*Two-chamber view longitudinal strain (%)*				
Basal inferior	14.9 ± 4.3 (8–25.5)	19.9 ± 4.5 (13–26)	18.1 ± 5.8 (11–27)	0.030
Mid inferior†	14.2 ± 5.9 (5–33)	14.9 ± 2.9 (10–20.3)	15.6 ± 4.8 (9–24)	0.816
Apical inferior	13.6 ± 5.5 (6–25)	12.7 ± 4.9 (2.5–18)	13.2 ± 4.8 (8–22)	0.900
Basal anterior†	17.8 ± 3.7 (12–27.3)	19.6 ± 4.4 (13.3–26)	18.7 ± 5.2 (11–25)	0.564
Mid anterior†	17.2 ± 5.6 (9–36)	16.6 ± 3.3 (11–21)	16.1 ± 5.4 (11.9–28)	0.854
Apical anterior†	13.8 ± 4.6 (2–22.3)	14.3 ± 3.9 (10–22)	13.8 ± 6 (6.6–26)	0.955
GLS 2ChS (%)*†	15.6 ± 3 (9.3–20.4)	16.9 ± 2 (12.5–19)	16.1 ± 4.3 (12–25.3)	0.597
*Three-chamber view longitudinal strain (%)*				
Basal inferior lateral†	21.6 ± 6.2 (12.4–43)	22.9 ± 6.8 (12–36)	18.2 ± 9.7 (5–32)	0.389
Basal anterior septal†	22.4 ± 7.2 (10.5–49)	19.8 ± 7.4 (10.3–35)	21.2 ± 5.2 (12.5–26)	0.633
Mid inferior lateral†	15 ± 3.9 (9.1–25)	18 ± 3.1 (12.5–21.3)	13.7 ± 5.5 (5–24)	0.095
Mid anterior septal†	18.9 ± 4.2 (8–24.8)	19.6 ± 4.8 (9–24)	18.1 ± 4.4 (11–24)	0.782
Apical lateral†	13.9 ± 4.8 (5.5–22)	16.8 ± 5.5 (7–26)	13.7 ± 2.3 (11–18)	0.267
Apical septal†	18.4 ± 5.1 (6–25)	18.6 ± 4.2 (11.4–24)	16.1 ± 6.3 (5–22.7)	0.522
GLS 3ChS (%)†	18.1 ± 2.6 (14–22.3)	19.5 ± 4 (11.4–24)	17.1 ± 4.4 (9–22)	0.342
Total GLS†	16.7 ± 2.3 (10–20.7)	18.7 ± 2.1 (15–21)	16.9 ± 3.3 (10–21)	0.131

AHA, American Heart Association; GLS 4ChS, global longitudinal strain measured at four-chamber ultrasonic view obtained from speckle-tracking echocardiography; GLS 2ChS, global longitudinal strain measured at two-chamber ultrasonic view obtained from speckle-tracking echocardiography; GLS 3ChS, global longitudinal strain measured at three-chamber ultrasonic view obtained from speckle-tracking echocardiography; EDV, end-diastolic volume; ESV, end-systolic volume; LVEF, left ventricular ejection fraction. Analyses were done for comparing antecedent Kawasaki patients and controls and also patients without coronary aneurysm and controls.

# Levy et al. Reference Ranges of Left Ventricular Strain Measures by Two-Dimensional Speckle-Tracking Echocardiography in Children: A Systematic Review and Meta-Analysis. Journal of the American Society of Echocardiography 2016; 29:209–25.

*: p ≤ 0.05 level for antecedent Kawasaki patients with unaffected coronary arteries and patients with coronary aneurysm.

†: The statistical difference was not significant (p > 0.05) for coronary aneurysm (−) versus coronary dilatation in the Kawasaki disease group.

§: The statistical difference was significant for patients with coronary dilatation and coronary aneurysm (p ≤ 0.05).

Figure. Strain measurements of cardiac segments on speckle tracking echocardiography (STE). A. Strain measurements on speckle tracking echocardiography in 4-chamber view. B. Supplying segments of coronary arteries on 4-chamber view.
